# Cerebral autoregulation monitoring in neonates and infants after cardiac surgery with cardiopulmonary bypass - comparison of single ventricle and biventricular physiology

**DOI:** 10.3389/fped.2025.1540870

**Published:** 2025-03-28

**Authors:** Marcel Methner, Bernadett Schwaneberg, Berit Wikidal, Simon Schmid, Julian Zipfel, Maximilian Iller, Martin U. Schuhmann, Yordan H. Georgiev, Harry Magunia, Rafal Berger, Christian Schlensak, Johannes Nordmeyer, Felix Neunhoeffer

**Affiliations:** ^1^ Department of Paediatric Cardiology, Pulmology and Paediatric Intensive Care Medicine, University Children’s Hospital of Tuebingen, Tuebingen, Germany; ^2^ Section of Paediatric Neurosurgery, Department of Neurosurgery, University Hospital Tuebingen, Tuebingen, Germany; ^3^ Department of Anaesthesiology and Intensive Care Medicine, University Hospital Tuebingen, Tuebingen, Germany; ^4^ Department of Thoracic and Cardiovascular Surgery, University Hospital Tuebingen, Tuebingen, Germany

**Keywords:** cerebral autoregulation, cardiopulmonary bypass, near-infrared spectroscopy, cardiac surgery, congenital heart disease, critical care

## Abstract

**Introduction:**

Cardiopulmonary bypass surgery can lead to impaired cerebral autoregulation with the risk for ischemia, hemorrhage and delirium. In particular, infants with single ventricle physiology have altered hemodynamics with persistent veno-arterial shunting, cyanosis and diastolic run-off after surgery, which may have negative effects on cerebral autoregulation.

**Methods:**

Cerebral autoregulation was prospectively monitored in 83 neonates and infants after open heart surgery with cardiopulmonary bypass after admission to the pediatric intensive care unit. Autoregulation indices were determined using near-infrared spectroscopy, correlating regional cortical oxygen saturation and local hemoglobin levels with invasive mean arterial pressure. Intact autoregulation was defined as cerebral oxygenation index (COx) < 0.4 and hemoglobin volume index (HVx) < 0.3. A subgroup analysis was performed between 55 infants after biventricular repair surgery and 15 infants after palliative surgery.

**Results:**

The mean lower limit of autoregulation was 46 ± 6 mmHg (COx) and 46 ± 7 mmHg (HVx). The optimal mean arterial pressure according to cerebral autoregulation was 56 ± 8 mmHg (COx) and 55 ± 7 mmHg (HVx). The mean upper limit was 65 ± 9 mmHg (COx) and 65 ± 8 mmHg (HVx). Intact autoregulation occurred during 84 ± 8% (COx) and 77 ± 10% (HVx) of the monitored time. No significant differences were found in autoregulation parameters between single ventricle and biventricular groups. However, the single ventricle group had significantly lower arterial and cerebral oxygen saturation.

**Discussion:**

A standardized blood pressure management may not be sufficient in children after cardiac surgery due to potentially impaired cerebral autoregulation. Therefore, cerebral autoregulation monitoring should be considered in this patient population.

## 1 Introduction

Despite improved survival rates in children with congenital heart disease, many patients experience impaired neurological outcomes. Dysfunctional autoregulation can result in the loss of this protective mechanism, increasing the risk of hyper- and hypoperfusion, which may lead to ischemia, hemorrhage, and delirium. Infants with congenital heart disease who undergo cardiac surgery with cardiopulmonary bypass and require intensive care are at a cumulative risk for impaired autoregulation. Several factors contributing to potential brain injury in these patients – such as altered hemodynamics, hypoxemia, increased metabolic demands, and the inflammatory response triggered by cardiopulmonary bypass (CPB) surgery – have been discussed (1–3). Cerebral autoregulation exhibits an autoregulatory plateau in which cerebral blood flow is constant relative to mean arterial pressure (MAP). The autoregulatory plateau is limited by the lower limit of autoregulation (LLA) and the upper limit of autoregulation (ULA). Beyond these limits, cerebral blood vessels become pressure passive and cerebral blood flow cannot be maintained (4, 5). Efforts to improve neurological outcomes include non-invasive neuromonitoring of cerebrovascular autoregulation using near-infrared spectroscopy (NIRS) to measure regional cerebral oxygen saturation (cSO_2_) and relative tissue hemoglobin concentration (rTHb). By correlating with MAP, the autoregulation indices cerebral oximetry index (COx) and hemoglobin volume index (HVx) can be determined and cerebral autoregulation can be mapped (4, 6). Intact autoregulation shows minimal correlation between cSO_2_ or rTHb and MAP, while impaired autoregulation shows greater correlation, indicating higher indices. The MAPopt reflects the optimal blood pressure for stable cerebral autoregulation (4, 6).

Current blood pressure guidelines are largely based on empirical data, which inadequately define optimal management strategies for cerebral perfusion and the best blood pressure approach for brain, visceral organs, and cardiac benefits (7–9). Children with single ventricle physiology face higher risks due to increased mortality, intensive care complications, and surgical severity compared to biventricular repair. After stage 1 palliation in single ventricle physiology, the systemic and pulmonary circulations are parallel. The single ventricle perfuses the systemic circulation via the neoaorta and the pulmonary circulation via a central or Blalock-Taussig shunt. The presence of a shunt may result in diastolic run-off into the pulmonary circulation, leading to reduced diastolic blood flow and pressure in the systemic circulation. This may contribute to persistent postoperative cyanosis, which is suspected to affect cerebral autoregulation (10–12). Only children with a central or Blalock-Taussig shunt were included in these analyses; right ventricle-pulmonary artery shunts were not performed.

The aim of this study was to determine postoperative cerebral autoregulation parameters using NIRS and ICM+ software (Cambridge Enterprises, UK) and further to identify possible differences in children with single ventricle physiology compared to surgery resulting in biventricular repair.

## 2 Methods

In this prospective, nonrandomized, monocentric study, 83 neonates and infants with congenital heart disease underwent cardiac surgery with CPB and were admitted to the pediatric intensive care unit at the University Hospital of Tuebingen, Germany, from January 2019 to July 2023. Inclusion criteria were age less than one year and CPB surgery with parental consent obtained. Ethical approval was granted (project number 763/2016BO1) and the study was conducted in accordance with the Declaration of Helsinki. Post-surgery, autoregulation monitoring began in the intensive care unit, where standard treatment protocols included a target mean arterial pressure (MAP) >45 mmHg for children under 6 months and >50 mmHg for older children.

Children were divided into four subgroups: (1) single ventricle physiology with postoperative stage 1 palliation, (2) biventricular repair, (3) Glenn surgery, and (4) other surgeries (e.g., atrioseptectomy or aortic arch reconstruction). Although children after Glenn surgery still have a single-ventricle physiology, they were categorized as a separate group because they no longer have parallel circulation and have increased upper body venous congestion, which can affect cerebral autoregulation, compared to children after stage 1 palliation. Due to the small sample size and heterogeneity of the patient cohort, all children who underwent cardiac surgery with cardiopulmonary bypass were analyzed together to obtain the most comprehensive data set possible. However, because some subgroups were very small, a detailed analysis was performed only between group 1 and group 2.

### 2.1 NIRS measurement

Cerebral autoregulation was evaluated using a near-infrared spectroscopy (NIRS) device (INVOS™ 5100C Cerebral/Somatic Oximeter, Medtronic, USA) equipped with an OxyAlert™ NIRSensor. The system utilizes a two-wavelength LED source (730 and 810 nm) and two photodiode detectors positioned at source-detector separations of 30 and 40 mm. Measurements of regional cerebral oxygen saturation (rSO_2_) and relative tissue hemoglobin (rTHb) were obtained via a sensor placed on the forehead, lateral to the midline, with continuous data acquisition facilitated by dedicated software (ICM+, Cambridge Enterprises, UK) running on a bedside laptop (13). Oxygenated and deoxygenated hemoglobin have different light absorption characteristics. Near-infrared spectroscopy uses this difference to measure oxygenation levels. At a specific wavelength, known as the isosbestic point, both types of hemoglobin exhibit the same absorption. By using two wavelengths – one where they differ and one at the isosbestic point – this method determines regional oxygen saturation (cSO_2_) and the relative tissue hemoglobin concentration (rTHb), which includes both oxygenated and deoxygenated hemoglobin. The hemoglobin volume index (HVx) was derived using a continuous moving Pearson correlation between MAP and rTHb, which remains unaffected by fluctuations in oxygen saturation. HVx is based on the premise that autoregulatory vasoconstriction and vasodilation result in cerebral blood volume changes proportional to variations in relative tissue hemoglobin. Similarly, the cerebral oxygenation index (COx) was calculated by determining the correlation coefficient between MAP and rSO_2_, as previously described (14).

**Figure 1:**
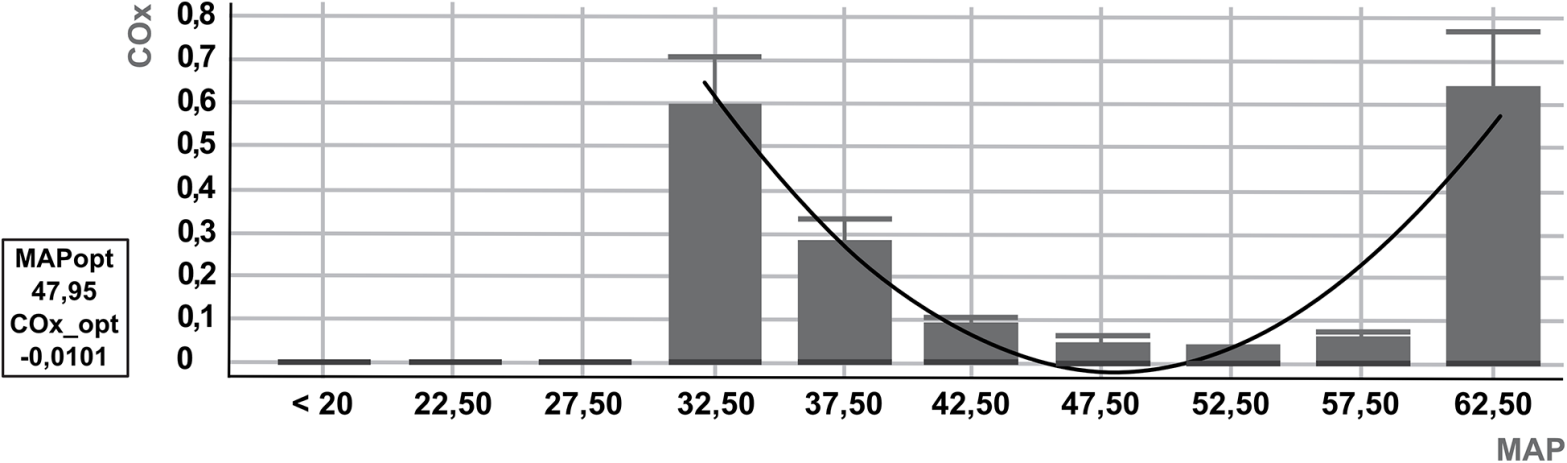
Example U-curve of cerebral autoregulation by ICM+. U-curve based on cerebral oximetry index (COx). The optimal mean arterial pressure according to cerebral autoregulation (MAPopt) is 47.95, defined as the lowest COx (COx_opt: −0.0101). Below and above MAPopt, the U-curve rises towards the COx cutoff of 0.4, which defines the lower and upper limits of autoregulation (LLA = 35 mmHg, ULA = 61 mmHg).

Both indices were calculated using a 300-s analysis window, with Pearson correlation coefficients applied. COx and HVx values were binned into 5 mmHg increments of mean arterial pressure, and bar graphs were generated accordingly. Monitor data were recorded at a sampling rate of 100 Hz (13). Autoregulation parameters were evaluated after artifact removal, excluding a 10-min period of implausible data, such as those caused by arterial blood gas sampling, to ensure accurate measurements. Autoregulation parameters were then determined by viewing a U-curve generated by the ICM+software for HVx and COx (Figure 1). The optimal MAP according to cerebral autoregulation (MAPopt) is displayed numerically by the ICM+ software and represents the lowest point of the U-curve, corresponding to the blood pressure range with the lowest autoregulation indices (4, 5). HVx and COx are correlation coefficients and range from −1 to +1. LLA and ULA were defined as the blood pressure range with an excess of HVx > 0.3 (6, 15) and COx > 0.4 (4). Intact autoregulation is therefore defined as HVx <0.3 or COx <0.4. The ICM+ software can be used to define the percentage of time with intact autoregulation. The most plausible U-curve was retrospectively selected based on the earliest feasible time point during the measurement period. Initially, an 8-hour interval was used to visualize the U-curves. If no clear U-curve could be identified, the interval was gradually reduced to a minimum of 3 h. In cases where autoregulation parameters remained stable throughout the entire measurement period, these values were included in the analysis. If additional distinct autoregulatory ranges were identified later in the recording that differed significantly from the initially observed range, these were also identified and documented.

### 2.2 Data collection

General patient data such as age, weight, sex, and surgery performed were obtained from the ISH program (SAP, Germany). Vital signs, arterial blood gas analysis, and ventilation parameters were obtained from the IntelliSpace Critical Care and Anesthesia ICCA hospital information system (Philips, Germany). The following parameters were analyzed: CBP duration, hemoglobin concentration (Hb), arterial partial pressure of oxygen (paO_2_), arterial partial pressure of carbon dioxide (paCO_2_), arterial oxygen saturation (aSO_2_), lactate concentration, norepinephrine consumption, epinephrine consumption, milrinone consumption, arterial oxygen content (caO_2_); calculated as caO_2_ [ml/dl] = aSO_2_ × Hb × 1.34 + paO_2_ × 0.0031, and cerebral oxygen content (ccO_2_); calculated as ccO_2_ [ml/dl] = cSO_2_ × Hb × 1.34 + paO_2_ × 0.0031. The difference between caO_2_ and ccO_2_ was used to calculate the arterio-cerebral oxygen content difference (acDO_2_) [ml/dl]. Cerebral fractional tissue oxygen extraction (cFTOE) as a parameter of cerebral oxygen extraction was calculated as cFTOE = (aSO_2_ − cSO_2_)/aSO_2_. Risk adjustment was assessed according to the RACHS-1 (Risk Adjustment for Congenital Heart Surgery) classification. The vasoactive-inotropic score (VIS), which combines the catecholamines used into one score, was calculated as VIS = dopamine dose (μg/kg/min) + dobutamine dose (μg/kg/min) + 100 × epinephrine dose (μg/kg/min) + 10 × milrinone dose (μg/kg/min) + 10,000 × vasopressin dose (units/kg/min) + 100 × norepinephrine dose (μg/kg/min). Lactate, aSO_2_, paO_2_, paCO_2_, Hb, cFTOE, caO_2_, ccO_2_, and acDO_2_ are baseline values. Norepinephrine, epinephrine, milrinone and VIS were measured at the beginning and end of the measurement period and are presented as averages. CSO_2_, rTHb, MAP, COx and HVx were measured every minute and are presented as averages. The mean time of measurement was 27 h after admission to intensive care unit.

### 2.3 Statistics

Statistical and graphical analyses were performed using SigmaPlot (Systat Software Inc., CA, USA). Data are presented as mean ± standard deviation and range. Parametric variables with normal distribution and equal variances were analyzed using either unpaired or paired t-test. Otherwise, the Mann–Whitney U test was used. Nominal variables were evaluated using Fisher's exact test. A *p*-value of < .05 was considered statistically significant.

## 3 Results

A total of 83 children, 49 boys and 34 girls, were included. Group 1 included 15 (18.1%) children with single ventricle physiology (SVP) after stage 1 palliation. Group 2 included 55 (66.3%) children with biventricular repair (biventricular physiology, BVP). Group 3 included 6 (7.2%) children after Glenn operation and group 4 included 7 (8.4%) children. In the case of ECMO therapy immediately after surgery, autoregulation monitoring was started after the end of ECMO therapy. We performed central ECMO therapy with CPB circulation. The mean duration of ECMO therapy was 24 h. The following malformation syndromes were present in the cohort: 8 (9.6%) trisomy 21, 3 (3.6%) VACTERL syndrome, 1 (1.2%) trisomy 18, 1 (1.2%) Alagille syndrome, 1 (1.2%) 22q11.2 deletion syndrome, 3 (3.6%) syndromes were not yet definitively diagnosed at the time of measurement.

The cardiac defects leading to surgery in the first group were as follows: 11 (73.3%) Norwood procedures, 3 (20.0%) Blalock-Taussig shunt dilation with valve correction, and 1 (6.7%) central aortopulmonary shunt. In group 2, who received biventricular correction, there were 17 (20.4%) ventricular septal defect, 9 (10.8%) tetralogy of Fallot, 8 (9.6%) transposition of the great arteries (TGA), 8 (9.6%) atrioventricular septal defects, 5 (6.0%) double outlet right ventricle, 4 (4.8%) truncus arteriosus communis, 3 (3.6%) coarctation of the aorta/aortic arch hypoplasia, 1 (1.2%) total anomalous pulmonary venous return. Group 3 included 6 (100%) children after Glenn operation. Group 4 included 2 (28.6%) left coronary artery translocations, 1 (14.3%) pulmonary artery dilation, 1 (14.3%) bipulmonary banding with atrioseptectomy, 1 (14.3%) complex aortic arch reconstruction, 1 (14.3%) artiostectomy, 1 (14.3%) complex bipulmonary banding with aortic arch reconstruction.

### 3.1 Clinical parameter and autoregulation parameter

**Figure 2:**
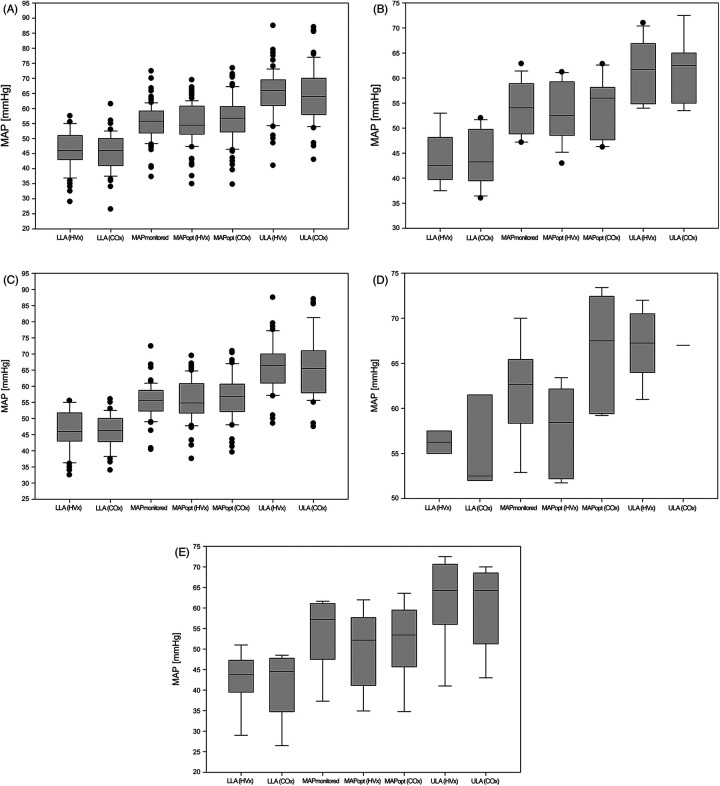
Autoregulation parameters. **(A)** all children (groups 1-4); **(B)** single ventricle physiology (group 1); **(C)** biventricular physiology (group 2); **(D)** Glenn surgery (group 3); **(E)** other surgeries (Group 4). COx, cerebral oximetry index; HVx, hemoglobin volume index; LLA, lower limit of autoregulation; MAP, mean arterial pressure; MAPmonitored, MAP recorded every minute during the measurement period; MAPopt, optimal MAP according to cerebral autoregulation; ULA, upper limit of autoregulation.

Table 1 shows the patient characteristics and clinical parameters, Table 2 shows the autoregulation parameters of all 83 children, group 1 and group 2. Figure 2 shows the autoregulation parameters of all 83 children and those for each subgroup. HVx determined the LLA in 66% and COx determined the LLA in 76%, while HVx determined the ULA in 78% and COx determined the ULA in 62%. MAPopt was determined in 95% of cases with HVx and COx. Note the narrow autoregulatory range of 19 ± 6 mmHg (9–37 mmHg) based on HVx and 19 ± 7 mmHg (6–38 mmHg) based on COx as the difference between LLA and ULA, i.e., the area of intact autoregulation. As well as the small lower limit reserve as the difference between MAPopt and LLA with 10 ± 4 mmHg (4–30 mmHg) based on HVx and 11 ± 4 mmHg (3–22 mmHg) based on COx. There were no significant differences between HVx and COx based autoregulation parameters (*P* < 0.05).

**Table 1:** Clinical and autoregulation parameters.

Clinical and autoregulation parameters	All children group 1–4 (*n* = 83)	Single ventricle physiology (*n* = 15)	Biventricular physiology (*n* = 55)	*p*-value
Age [d]	112 ± 85 (4–309)	63 ± 77 (5–226)	126 ± 84 (7–109)	**0** **.** **02**
Male gender	50	10	28	0.38
Weight [kg]	5.0 ± 1.8 (2.1–9.6)	4.2 ± 1.6 (2.6–7.1)	5.2 ± 1.8 (2.2–9.6)	0.07
Neonates (age <28 days)	25	8	13	0.05
Body surface area [qm]	0.27 ± 0.07 (0.16–0.44)	0.24 ± 0.07 (0.18–0.36)	0.28 ± 0.06 (0.16–0.44)	0.07
RACHS-Score	3.4 ± 1.3 (2–6)	5.1 ± 1.4 (3–6)	3 ± 1 (2–4)	**<0.001**
Measuring time [h]	27 ± 13 (2–51)	31 ± 13 (7–51)	25 ± 12 (2–51)	**0**.**04**
CPB duration [min]	126 ± 145 (17–279)	125 ± 50 (48–248)	119 ± 51 (17–279)	0.36
VA-ECMO	3	2	1	0.11
aSO_2_ [%]	92 ± 9 (67–100)	80 ± 8 (67–96)	97 ± 4 (78–100)	**<0**.**001**
paO_2_ [mmHg]	88 ± 39 (246–33)	46 ± 10 (33–79)	106 ± 34 (44–246)	**<0**.**001**
paCO_2_ [mmHg]	43 ± 6 (31–56)	47 ± 5 (38.2–54.4)	43 ± 6 (31–56)	**0**.**03**
Hb [g/dl]	12.9 ± 2.2 (8.7–20)	13.9 ± 2.1 (9.2–16.9)	12.3 ± 1.9 (8.7–16.6)	**0**.**01**
Lactate [mmol/L]	1.6 ± 1 (0.4–5.1)	1.9 ± 1 (0.8–4.7)	1.6 ± 0.9 (0.4–5.1)	0.25
VIS	8.3 ± 5 (0.2–27)	8 ± 4.7 (0.5–19.7)	9.1 ± 5.2 (0.2–27)	0.97
cFTOE	0.31 ± 0.12 (0.04–0.58)	0.28 ± 0.12 (0.12–0.53)	0.31 ± 0.12 (0.04–0.58)	0.26
caO_2_ [ml/dl]	16.1 ± 2.7 (10.5–23.3)	15.1 ± 2.7 (10.5–19.5)	16.2 ± 2.5 (11.7–22.6)	0.13
ccO_2_ [ml/dl]	11.2 ± 2.8 (5–20.3)	11.1 ± 3.1 (5–15.4)	11.1 ± 2.9 (6.1–20.3)	0.47
acDO_2_ [ml/dl]	4.9 ± 3 (0.8–10.1)	4 ± 1.4 (1.9–6.1)	5.1 ± 1.9 (0.8–9.6)	0.05
cSO_2_ [%]	65 ± 9 (38–87)	59 ± 9 (40–76)	67 ± 9 (49–87)	**0**.**004**
rTHb	0.4 ± 0.8 (−1.6–2)	0.5 ± 0.7 (−0.7–1.3)	0.3 ± 0.8 (−1.6–2)	0.53
MAP [mmHg]	55 ± 6 (37–72)	54 ± 5 (47–63)	55 ± 6 (40–72)	0.36
COx	−0.02 ± 0.19 (−1.38–0.4)	0.01 ± 0.09 (−0.21–0.17)	0.01 ± 0.11 (−0.23–0.4)	0.99
HVx	−0.05 ± 0.2 (−1.1–0.31)	−0.06 ± 0.08 (−0.18–0.06)	−0.03 ± 0.19 (−1.01–0.31)	0.29

Analysis included data from all groups (1–4), but subgroup analysis focused on group 1 (single ventricle physiology) and group 2 (biventricular repair).

Bold values indicate statistical significance at *p* < 0.05.

acDO_2_, arterio-cerebral oxygen content difference; aSO_2_, arterial oxygen saturation; caO_2_, arterial oxygen content; ccO_2_, cerebral oxygen content; cFTOE, cerebral fractional tissue oxygen extraction; COx, cerebral oximetry index; cSO_2_, cerebral oxygen saturation; Hb, hemoglobin concentration; HVx, hemoglobin volume index; MAP, mean arterial pressure; paCO_2_, arterial partial pressure of carbon dioxide; paO_2_, arterial partial pressure of oxygen; RACHS score, Risk Adjustment for Congenital Heart Surgery score; rTHb, relative tissue hemoglobin concentration; VA-ECMO, veno-arterial extracorporeal membrane oxygenation; VIS, vasoactive-inotropic score.

**Table 2:** Autoregulation parameters.

Autoregulation parameters	All children group 1–4 (*n* = 83)	Single ventricle physiology (*n* = 15)	Biventricular physiology (*n* = 55)	*p*-value
LLA HVx (mmHg)	46 ± 7 (29–58)	44 ± 6 (38–53)	46 ± 6 (33–56)	0.34
LLA COx (mmHg)	46 ± 6 (27–62)	44 ± 6 (36–52)	46 ± 5 (34–56)	0.25
MAPopt HVx (mmHg)	55 ± 7 (35–70)	53 ± 6 (43–61)	56 ± 6 (38–70)	0.25
MAPopt COx (mmHg)	56 ± 8 (35–74)	54 ± 6 (46–63)	57 ± 7 (40–71)	0.29
MAPmonitored (mmHg)	55 ± 6 (37–72)	54 ± 5 (47–63)	55 ± 6 (40–72)	0.36
ULA HVx (mmHg)	65 ± 8 (41–88)	61 ± 6 (54–71)	66 ± 8 (49–88)	0.08
ULA COx (mmHg)	65 ± 9 (43–87)	61 ± 6 (54–73)	66 ± 9 (48–87)	0.13
Percentage of time with HVx <0.3 in %	77 ± 10 (42–95)	80 ± 5 (72–91)	77 ± 11 (42–95)	0.72
Percentage of time with COx <0.4 in %	84 ± 8 (43–95)	83 ± 6 (73–93)	83 ± 9 (43–95)	0.35

Analysis included data from all groups (1–4), but subgroup analysis focused on group 1 (single ventricle physiology) and group 2 (biventricular repair).

COx, cerebral oximetry index; HVx, hemoglobin volume index; LLA, lower limit of autoregulation; MAP, mean arterial pressure; MAPmonitored, average MAP measured every minute during measurement; MAPopt, optimal MAP regarding to intact cerebral autoregulation; ULA, upper limit of autoregulation.

### 3.2 Dynamics of autoregulation parameters

In 29% (*n* = 24), autoregulation parameters were dynamic over time and at least a second autoregulation range was identified. The LLA (HVx) of the first autoregulation range was 44 ± 6 mmHg (34–55 mmHg) vs. 51 ± 4 mmHg (44–56 mmHg) of the second autoregulation range. The LLA (COx) of the first autoregulation range was 44 ± 6 mmHg (36–55 mmHg) vs. 48 ± 6 mmHg (41–58 mmHg) of the second autoregulation range. The first MAPopt (HVx) was 51 ± 6 mmHg (38–61 mmHg) vs. 60 ± 7 mmHg (42–74 mmHg) and the first MAPopt (COx) was 52 ± 7 mmHg (40–62 mmHg) vs. 60 ± 7 mmHg (46–71 mmHg) of the second range. The first ULA (HVx) was 59 ± 6 mmHg (49–68 mmHg) vs. 69 ± 10 mmHg (50–81 mmHg) and the ULA (COx) was 60 ± 7 mmHg (48–69 mmHg) vs. 70 ± 12 mmHg (52–92 mmHg). All differences between autoregulation ranges were significant at *P* < 0.05.

### 3.3 Comparison of single ventricle and biventricular physiology

There was no significant difference in the autoregulation parameters between the two groups. There was no significant difference in the lower limit reserve [*P* = 0.2 (HVx), *P* = 0.19 (COx)] and the area of the autoregulatory plateau [*P* = 0.17 (HVx), *P* = 0.22 (COx)]. No significant correlation was found between cSO_2_ and intact autoregulation in either the palliation group [*r* = −0.42, *P* = 0.12 (HVx); *r* = −0.3, *P* = 0.27 (COx)] or the correction group [*r* = −0.17, *P* = 0.12 (HVx); *r* = 0.11; *P* = 0.31 (COx)].

## 4 Discussion

**Figure 3:**
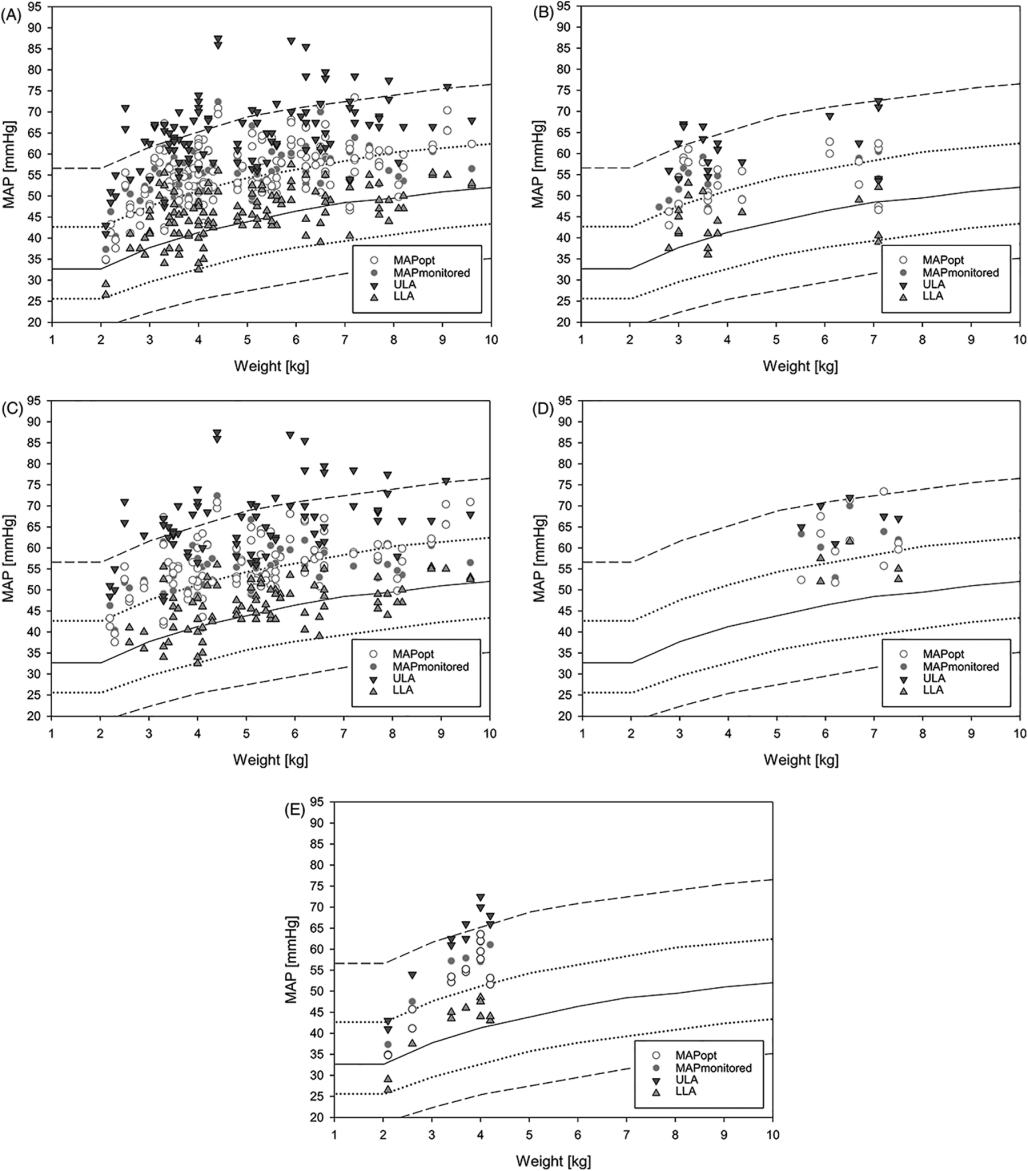
Plot of all measured LLA, MAPmonitored, MAPopt and ULA. **(A)** all children (groups 1–4); **(B)** single ventricle physiology (group 1); **(C)** biventricular physiology (group 2); **(D)** Glenn surgery (group 3); **(E)** other surgeries (Group 4). The empirical MAP during general anesthesia from de Graaff (7) is plotted in the background, with the continuous line showing the mean MAP and the discontinuous line showing the first and second standard deviations. LLA, lower limit of autoregulation; MAP, mean arterial pressure; MAPmonitored, MAP recorded every minute during the measurement period; MAPopt, optimal MAP according to cerebral autoregulation; ULA, upper limit of autoregulation.

In this study, mean measured MAP and retrospectively determined MAPopt exceeded empirical MAP values by approximately 10 mmHg in healthy children under general anesthesia (Figure 3) (7). Looking at the average MAPmonitored and MAPopt, there seems to be little difference between them. The MAPmonitored is mostly within the LLA and ULA. However, this is the average MAP over the entire measurement period. Therefore, there may still be relevant under- or overshoots of autoregulation limits. Intact cerebral autoregulation was observed on average only in 77% ± 10% (42%–95%) (HVx) and 84% ± 8% (43%–95%) (COx) of the measured time. With the knowledge of the MAPopt and the autoregulatory limits, a precise and targeted blood pressure therapy could be performed and an exceeding or falling below the autoregulatory limits could be avoided and the percentage of impaired autoregulation could be reduced. However, the impact of this on patient outcome has not yet been studied. The narrow margin between LLA and empirical MAP suggests a risk of cerebral hypoperfusion, as empirical MAP is often below LLA (16). The narrow autoregulatory plateau between LLA and the ULA highlights the limited safe blood pressure range for these patients. Compared to the autoregulatory range of approximately 100 mmHg in healthy adults (17), blood pressure management in this vulnerable population is particularly challenging.

Pezzato et al. monitored 28 postoperative neonates with hypoplastic left heart syndrome and neonates with TGA using NIRS and ICM+ software and found similar autoregulatory parameters as in our study. They found a mean LLA of 48 mmHg [interquartile range (IQR): 43–50], MAPopt of 55 mmHg (IQR: 53–58), ULA of 64 mmHg (IQR: 60–66), and an autoregulatory plateau of 16 mmHg (IQR: 13–20). The autoregulatory plateau was significantly narrower 24–48 h postoperatively compared to earlier and later periods. The range of data was narrower compared to our study, possibly due to greater homogeneity in the subgroups of Pezzato et al. (18). Cabrera et al. also found a mean MAPopt of 55 mmHg (51–64 mmHg) based on HVx in infants after Glenn surgery (19).

The risk of cerebral ischemia and periventricular leukomalacia increases with the degree and duration of hypotension below the LLA (20–24), while hypertension above the ULA may increase the risk of cerebral hyperperfusion, delirium, cerebral edema, and hemorrhage (25, 26). It remains unclear whether achieving normotension significantly improves neurological outcomes, as pharmacologic control, particularly above the ULA, may increase the risk of cerebral hemorrhage and cardiac damage (8, 27, 28). Similar to other studies (4, 5, 29), this study shows a high interindividual variability and a wide range of autoregulation parameters, indicating the need for individualized peri- and postoperative neuromonitoring, as predictive indicators seem unreliable (8, 30). Physiological goals must be considered when deciding on optimal blood pressure management. Although cerebral and renal perfusion may benefit from higher MAP, the increased systemic vascular resistance may place a strain on the operated heart (8, 22, 28, 31). In adults, prolonged MAP below the LLA during CPB surgery has been associated with an increased risk of acute renal failure, delirium, stroke, and prolonged mechanical ventilation (32).

Claessens et al. compared impaired autoregulation (COx >0.5) in 28 children with SVP, 19 with transposition of the great arteries (TGA) and 30 with left ventricular outflow tract obstruction (LVOTO). In their study, Claessens et al. observed impaired autoregulation in 15% ± 19% of the postoperative period using a COx >0.5 cutoff (33). In the present study, 77% ± 10% (42%–95%) (HVx) and 84% ± 8% (43%–95%) (COx) of the measured time were within the autoregulated range, indicating impaired autoregulation in an average of 23% (HVx) and 16% (COx) of the time, with considerable variability observed. Pezzato et al. found impaired cerebral autoregulation in 31.6% (IQR: 21.1–38.3) of the time in 28 neonates using a COx >0.3 cutoff, with the most dysregulation occurring 24–48 h postoperatively. Based on these data, cerebral autoregulation is most impaired and the autoregulatory plateau is narrowest 24–48 h postoperatively, suggesting this period is the most vulnerable for cerebral autoregulation (18). Despite different cut-offs, these results appear comparable across all three studies (18, 33).

In our study, 29% of infants had at least one second of significantly different autoregulation area. This intra-individual dynamic was also demonstrated by Montgomery et al. using a novel co-trending method to determine intraoperative LLA during CPB surgery. In adults, a mean change in LLA of 3 mmHg was observed on average 7 times (34). Pezzato et al. also observed dynamic autoregulation parameters in 28 neonates during the first 78 h postoperatively after palliation and corrective surgery (18). A one-time determination of autoregulation parameters and a fixed blood pressure strategy do not seem to be adequate. Further studies on the dynamics of autoregulation are necessary.

Children with SVP were younger and had more severe interventions and heart defects. As expected, a difference in arterial and cerebral oxygen levels was observed. The effect of diastolic run-off from the Blalock-Taussig shunt on cerebral perfusion, oxygenation, and autoregulation in patients with SVP is controversial (10–12). In our study, no effect of diastolic run-off on cerebral autoregulation limits or MAPopt was demonstrated. Similarly, Pezzato et al. found no significant differences in MAPopt (*P* = 0.3), LLA (*P* = 0.67), ULA (*P* = 0.14), or autoregulatory plateau (*P* = 0.19) between neonates with TGA and those with SVP (18). Claessens et al. found significantly more time with impaired autoregulation (COx >0.5) in children with SVP compared to those with TGA or left ventricular outflow tract obstruction (LVOTO) (*P* < 0.08). Of the 58 patients with SVP and LVOTO, 21 underwent deep hypothermic cardiac arrest (33). In our study, we could not demonstrate this difference, likely due to a relatively inhomogeneous comparison group and the absence of hypothermic cardiac arrest. Pezzato et al. also found no difference in impaired autoregulation between 7 Norwood and 21 arterial switch operations (32% vs. 30%, COx >0.3, *P* = 0.5) (18). Claessens et al. found a significant negative correlation between impaired autoregulation and cSO_2_ (*r* = −0.30, *P* = 0.01) (33). In our study, no significant correlation was observed between cSO_2_ and intact autoregulation in either the single ventricle or biventricular group. Additionally, Claessens et al. found no correlation between cSO_2_, FTOE, and intact autoregulation in new-onset brain injury. No difference in real-time measured MAP was found between the subgroups in our study or in the study by Claessens et al. (33). Neunhoeffer et al. found no differences in cFTOE or acDO_2_ between children who underwent palliative or repair surgery, similar to our findings (35).

It might be expected that in children with a single ventricle physiology, an increase in blood pressure would lead to an increase in oxygen saturation due to increased pulmonary perfusion. Given the positive correlation between arterial oxygen saturation (aSO_2_) and cerebral oxygenation (cSO₂) (36), significant fluctuations in blood pressure could potentially affect saturation levels and, consequently, autoregulation measurements based on cerebral oxygen saturation (COx). However, we were unable to demonstrate this effect as COx- and HVx-based autoregulation parameters did not differ significantly in children with single ventricle physiology.

Pezzato et al. showed that 7 out of 28 neonates (2 with single ventricle physiology and 5 after arterial switch operation) had acute neurological injury. These neonates had significantly higher COx values on average (*P* = 0.035), a higher proportion with COx >0.3 (39% vs. 29%, *P* = 0.017), and more time with MAP below the LLA (*P* = 0.048) compared to those without cerebral injury (18).

## 5 Limitations

Several limitations must be considered when interpreting our results. In some cases, mean arterial pressure (MAP) did not reach autoregulatory limits, preventing accurate determination of all autoregulatory parameters. Interventional control of MAP beyond these limits could be harmful, limiting the applicability of this method. In addition, periods without a plausible U-curve representation prevented autoregulation determination, posing a challenge for future interventional studies relying on real-time autoregulation data. The co-trending method proposed by Montgomery et al. offers promising advances for accurate real-time monitoring of cerebral autoregulation (33). In this study, autoregulation parameters were not explicitly analyzed based on specific time intervals, but the entire measurement period was evaluated. Dynamic changes in autoregulation parameters were determined and reported only when observed, without further investigation of potential influencing factors. Furthermore, the thresholds used to define intact autoregulation (COx <0.4, HVx <0.3) require further validation, especially in such a heterogeneous patient population. The lack of standardized cutoff values across different autoregulation studies further emphasizes the need for a more unified approach in future research. The small and heterogeneous patient cohort resulted in limited statistical power, particularly in subgroup analyses. To maximize data collection, all available patients were included, but due to the small sample size, only a comparative analysis between group 1 and group 2 was performed. The subgroups included cases with ECMO and malformation syndromes, reflecting the real-world diversity of this rare patient population and underscoring the complexity of cerebral autoregulation. Given its inherently variable nature, autoregulation is further influenced by multiple factors, including surgical techniques, shunting strategies, anesthetic protocols, use of vasoactive drugs, metabolic status, sedation levels, and pre-existing brain injury.

## 6 Conclusion

Blood pressure reference values for anesthetized children are largely empirical and often unsuitable for post-cardiac surgery patients. Cerebral autoregulation is individual and dynamic, making the prediction of optimal blood pressure based on pathophysiology or empirical data unsuitable for the individual child. No significant differences in autoregulation parameters were found between children with single ventricle physiology and biventricular physiology. Comprehensive neuromonitoring coupled with differentiated blood pressure management could help prevent hypo- or hyperperfusion, reduce catecholamine use, and potentially improve cardiac, renal, and cerebral perfusion. However, prioritizing neuroprotection is essential to improve outcomes in children. Currently, few studies have examined the relationship between postoperative cerebral autoregulation and neurological outcomes, highlighting the need for further research to determine whether actively optimizing cerebral autoregulation can lead to improved neurological outcomes.

## Data Availability

The raw data supporting the conclusions of this article will be made available by the authors, without undue reservation.
